# The Mutation of Rice MEDIATOR25, OsMED25, Induces Rice Bacterial Blight Resistance through Altering Jasmonate- and Auxin-Signaling

**DOI:** 10.3390/plants11121601

**Published:** 2022-06-17

**Authors:** Go Suzuki, Manatsu Fukuda, Nonawin Lucob-Agustin, Yoshiaki Inukai, Kenji Gomi

**Affiliations:** 1 Faculty of Agriculture, Kagawa University, Miki 761-0795, Japan; 2743001u@kagawa-u.ac.jp (G.S.); s22g641@kagawa-u.ac.jp (M.F.); 2 Philippine Rice Research Institute, Central Experiment Station, Science City of Muñoz 3119, Philippines; nonawinlucob@gmail.com; 3 International Center for Research and Education in Agriculture, Graduate School of Bioagricultural Sciences, Nagoya University, Nagoya 464-8601, Japan; inukaiy@agr.nagoya-u.ac.jp

**Keywords:** auxin, jasmonic acid, mediator, rice, *Xanthomonas oryzae* pv. *oryzae*

## Abstract

Rice bacterial blight disease caused by *Xanthomonas oryzae* pv. *oryzae* (*Xoo*) is one of the most severe diseases of rice. However, the regulatory mechanisms of rice defense against *Xoo* remain poorly understood. The rice MEDIATOR25, OsMED25—a subunit of the mediator multiprotein complex that acts as a universal adaptor between transcription factors (TFs) and RNA polymerase II—plays an important role in jasmonic acid (JA)-mediated lateral root development in rice. In this study, we found that OsMED25 also plays an important role in JA- and auxin-mediated resistance responses against rice bacterial blight. The *osmed25* loss-of-function mutant exhibited high resistance to *Xoo*. The expression of JA-responsive defense-related genes regulated by OsMYC2, which is a positive TF in JA signaling, was downregulated in *osmed25* mutants. Conversely, expression of some OsMYC2-independent JA-responsive defense-related genes was upregulated in *osmed25* mutants. Furthermore, OsMED25 interacted with some AUXIN RESPONSE FACTORS (OsARFs) that regulate auxin signaling, whereas the mutated osmed25 protein did not interact with the OsARFs. The expression of auxin-responsive genes was downregulated in *osmed25* mutants, and auxin-induced susceptibility to *Xoo* was not observed in *osmed25* mutants. These results indicate that OsMED25 plays an important role in the stable regulation of JA- and auxin-mediated signaling in rice defense response.

## 1. Introduction

Rice (*Oryza sativa* L.) is one of the most important crops worldwide and a model plant of monocotyledonous species with regard to molecular studies. Rice bacterial blight disease caused by the hemi-biotrophic pathogen *Xanthomonas oryzae* pv. *oryzae* (*Xoo*) is one of the most severe diseases of rice. *Xoo* is a vascular pathogen which remains within the xylem vessels throughout the course of the disease [1]. The two plant hormones, salicylic acid (SA) and jasmonic acid (JA), play important roles regarding resistance to biotrophic/hemi-biotrophic and necrotrophic pathogens in plants, respectively, but were suggested to exert mutually antagonistic interactions in dicotyledonous plants [2]. However, recent studies revealed that JA also plays an important role in the defense response to *Xoo* in rice [3]. The JA-responsive valine–glutamine (VQ)-motif-containing protein, OsVQ13, positively affects defense signaling regulated by OsWRKY45, which is a central transcription factor (TF) in the SA-mediated defense response to *Xoo* in rice [4,5].

The accumulation of bioactive JA, JA-isoleucine, induces upregulation of *OsWRKY45* and enhances resistance to *Xoo* in rice [6]. Additionally, many defense-related genes including *OsWRKY45* and *OsVQ13* are upregulated by both JA and the functional analogue of SA in rice, benzothiadiazole [5,7]. Furthermore, the rices ENHANCED DISEASE SUSCEPTIBILITY1 (OsEDS1) and PHYTOALEXIN DEFICIENT4 (OsPAD4), which are rice homologues of *Arabidopsis* EDS1 and PAD4, both regulate SA-dependent defense response in *Arabidopsis* but play important roles in the JA-mediated defense response to *Xoo* in rice [8]. JA biosynthesis and expression of JA-responsive defense-related genes are suppressed in rice after inoculation of the virulent *Xoo* [9,10]. Treatment with cell wall-degrading enzymes extracted from *Xoo* activates JA signaling and induces *Xoo* resistance in rice [11]. JA induces the production of antibacterial compounds that suppress *Xoo* growth [12,13]. The monoterpene compound, γ-terpinene, damages the plasma membranes of *Xoo* cells [14], and geraniol suppresses expression of cell-division-related genes in *Xoo* [15]. Conversely, linalool exerts no antibacterial effects on *Xoo*; however, it acts as a signaling molecule to induce *Xoo* resistance in rice [16]. These results suggest that the defense mechanism via JA and SA signaling in rice is notably different from that of *Arabidopsis*, and that JA plays a crucial role in resistance of rice to *Xoo*.

These intricate JA signaling networks in rice are strictly regulated by TFs. The basic helix-loop-helix (bHLH)-type of TFs, OsMYC2, OsbHLH034, and RICE EARLY RESPONSIVE TO JASMONATE1 (RERJ1) positively regulate JA-mediated defense response to *Xoo* in rice [17,18,19]. OsbHLH034 positively regulates biosynthesis of lignin, which is one of the most important components in the defense response to *Xoo* in rice [18,20,21]. RERJ1 interacts with OsMYC2 and positively regulates production of linalool by upregulating *linalool synthase* [19]. OsMYC2 is also activated under phosphate starvation conditions, resulting in the induction of resistance to *Xoo* in rice [22]. The OsMYC2 activity is suppressed by a complex that consists of rice JASMONATE ZIM (JAZ)-domain proteins (OsJAZs) and rice NOVEL INTERACTOR OF JAZ1 (OsNINJA1) [17,23]. The OsMYC2-interacting JAZ, OsJAZ8, acts as a repressor in the JA-induced defense response to *Xoo* in rice [24]. *OsNINJA1*-overexpressing rice plants are more susceptible to *Xoo*, due to the downregulation of OsMYC2-responsive defense-related genes [23]. The OsNINJA1-interacting protein OsSRO1a also acts as a mediator that suppresses OsMYC2-mediated defense response to *Xoo* in rice [25].

TFs, i.e., OsMYC2, do not directly interact with the general transcription machinery, including RNA polymerase II, during transcription. Rather, they bind to specific cis-acting sequences present in the promoter regions of target genes through their DNA-binding domains and recruit the MEDIATOR multiprotein complex which binds TFs to the RNA polymerase II [26]. The MEDIATOR complex contains numerous subunits, and approximately 50 subunits have been found in the rice genomes [27]. The MEDIATOR25 (MED25) subunit was first isolated as a regulator involved in the flowering process, and it also plays important roles in other plant growth processes regulated by several plant hormones, including JA [26,28]. Recently, we reported that *osmed25* loss-of-function rice mutants exhibited a JA-insensitive phenotype, and OsMED25 acts as a positive regulator in OsMYC2-mediated leaf senescence [29,30]. The OsMED25 directly interacts with OsMYC2 and positively regulates OsMYC2-responsive senescence-associated genes [30].

OsMED25 is involved in various JA signaling pathways; however, the involvement of OsMED25 in rice resistance to *Xoo* is unclear. In the present study, we investigated the role of OsMED25 in JA-mediated resistance to *Xoo* using an *osmed25* mutant. We also investigated the involvement of auxin in OsMED25-mediated resistance of rice to *Xoo*.

## 2. Results

### 2.1. Analysis of Xoo Resistance in osmed25 Mutants

**Figure 1:**
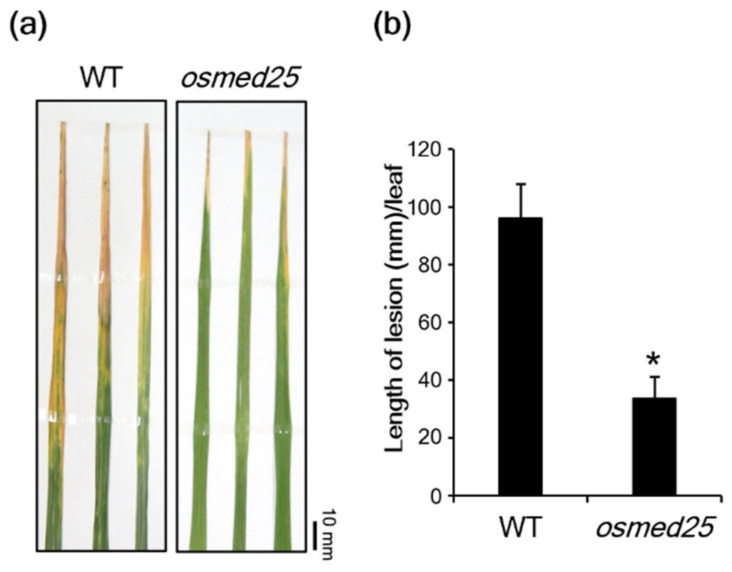
Enhanced resistance of *osmed25* mutants to *Xoo*. (**a**) Disease symptoms of bacterial blight in WT and *osmed25* mutant plants. Fifth-leaf blades were photographed 14 days after inoculation with *Xoo*. Scale bars = 10 mm. (**b**) Lengths of lesions on fifth-leaf blades 14 days after inoculation with *Xoo*. Data were analyzed using Student’s *t*-test (*n* = 13 for WT; *n* = 8 for *osmed25*). The asterisk indicates a significant difference from WT plants at *p* < 0.05.

Since we previously reported that OsMED25 is involved in root development and leaf senescence in rice through JA signaling [30], in the present study we investigated whether OsMED25 is involved in *Xoo* resistance. The *osmed25* mutants were inoculated with virulent *Xoo*, and lengths of blight lesions were measured two weeks after the inoculation. Blight lesions in *osmed25* mutants were significantly shorter than those in wild-type (WT) plants (Figure 1a,b).

### 2.2. Expression of JA-Responsive Defense-Related Genes in osmed25 Mutants

**Figure 2:**
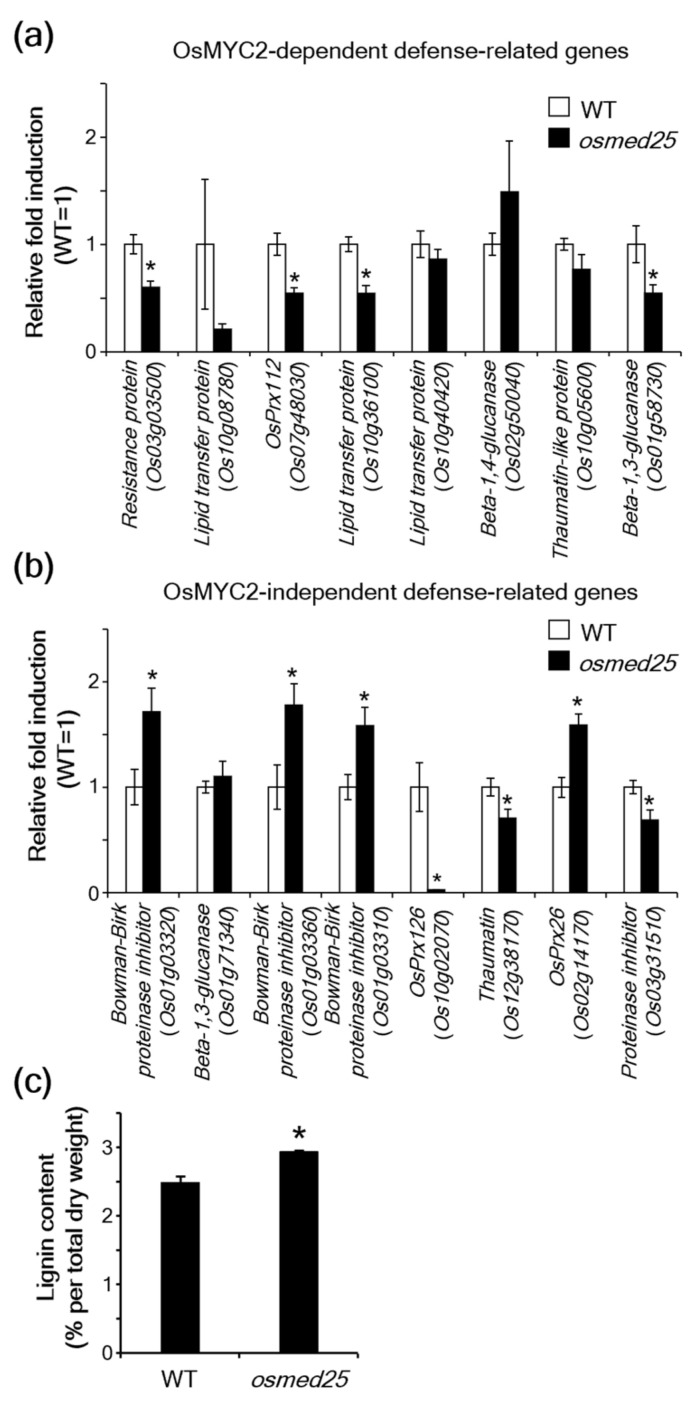
Expression of OsMYC2-dependent and -independent defense-related genes in *osmed25* mutants. (**a**) RT-qPCR analysis of OsMYC2-dependent defense-related genes in *osmed25* mutants. (**b**) RT-qPCR analysis of OsMYC2-independent defense-related genes in *osmed25* mutants. In (**a**) and (**b**), the values are means ± SE (*n* = 6). Data were analyzed using Student’s *t*-test. Asterisks indicate significant differences from WT plants at *p* < 0.05. (**c**) Lignin content in leaf blades of WT plants and *osmed25* mutants. Values are means ± SE (*n* = 5). Data were analyzed using Student’s *t*-test. The asterisk indicates a significant difference from WT plants at *p* < 0.05.

Because OsMED25 positively affected OsMYC2-mediated JA signaling in rice [30], we further investigated the expression of previously reported OsMYC2-responsive defense-related genes [17] in the *osmed25* mutants, which include *resistance protein* (*Os03g03500*), *lipid transfer protein* (*Os10g08780*), *peroxidase* (*OsPrx112*; *Os07g48030*), *lipid transfer protein* (*Os10g36100*), *lipid transfer protein* (*Os10g40420*), *beta-1,4-glucanase* (*Os02g50040*), *thaumatin-like protein* (*Os10g05600*), and *beta-1,3-glucanase* (*Os01g58730*). All the OsMYC2-responsive defense-related genes examined, except *beta-1,4-glucanase* (*Os02g50040*), tended to be downregulated in *osmed25* mutants compared to WT plants (Figure 2a).

We also investigated the expression of OsMYC2-independent JA-responsive defense-related genes in *osmed25* mutants. Those genes, which were identified in our previous studies [17,24], include the *Bowman–Birk proteinase inhibitor* (*Os01g03320*), *beta-1,3-glucanase* (*Os01g71340*), *Bowman–Birk proteinase inhibitor* (*Os01g03360*), *Bowman–Birk proteinase inhibitor* (*Os01g03310*), *peroxidase* (*OsPrx126*; *Os10g02070*), *thaumatin* (*Os12g38170*), *peroxidase* (*OsPrx26*; *Os02g14170*), and *proteinase inhibitor* (*Os03g31510*). Three OsMYC2-independent defense-related genes, i.e., *OsPrx126* (*Os10g02070*), *thaumatin* (*Os12g38170*), and *proteinase inhibitor* (*Os03g31510*), were significantly downregulated in *osmed25* mutants, whereas four, including three *Bowman–Birk proteinase inhibitors* (*Os01g03320*, *Os01g03360*, and *Os01g03310*) and *OsPrx26* (*Os02g14170*), were significantly upregulated in *osmed25* mutants compared to WT plants (Figure 2b). OsPrx26 is a putative class III peroxidase. Some class III peroxidases are thought to catalyze the polymerization of lignin precursors, such as monolignols [31], and lignin is known to play an important role in *Xoo* resistance [18,25]. Thus, we measured lignin content and found that lignin levels were significantly higher in *osmed25* mutants than in WT plants (Figure 2c).

### 2.3. Interactions between OsMED25 and OsARFs

**Figure 3:**
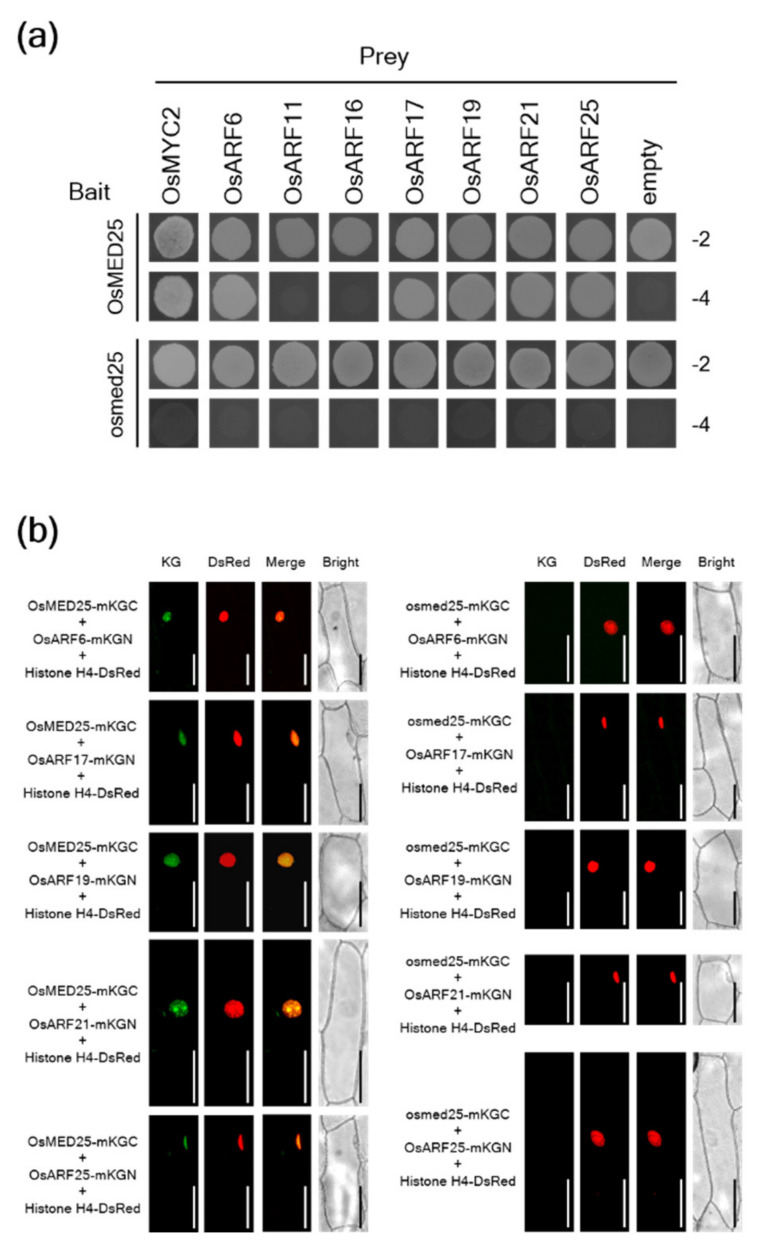
Interaction of OsMED25 with OsARFs. (**a**) Interaction of OsMED25 with OsARF proteins in yeast cells. The yeast strain AH109 carrying each construct was dropped on synthetic dropout (SD) glucose medium lacking Leu and Trp (−2) or on SD glucose medium lacking Ade, His, Leu, and Trp (−4). Images were recorded three days after dropping. (**b**) Interaction of OsMED25 with OsMED25-interacting OsARF proteins in plant cells. From left to right: KG, fluorescence images of KG protein; DsRed, fluorescence images of DsRed protein; Merge, overlap KG images and DsRed images; Bright, light-microscopy images. Scale bars = 100 µm.

In *Arabidopsis*, AtMED25 regulates auxin signaling through its interaction with AUXIN RESPONSE FACTORs (AtARFs), which are key TFs for the regulation of auxin responses in *Arabidopsis* [26]. The auxin signaling was reported to negatively regulate *Xoo* resistance in rice [32]. Thus, we investigated whether OsMED25 interacted with OsARFs. The rice genome contains 25 OsARFs [33], but OsARF6, OsARF11, OsARF16, OsARF17, OsARF19, OsARF21, and OsARF25 were selected as those possibly interacting with OsMED25 because they are considered to be transcriptional activators of auxin signaling [33]. OsMYC2 was used as a positive control regarding interactions with OsMED25 [30]. As shown in Figure 3a, OsMED25 interacted with OsARF6, OsARF17, OsARF19, OsARF21, and OsARF25 in yeast cells. These OsMED25-interacting OsARFs in yeast cells were further confirmed to be interacting with OsMED25 in plant cells (Figure 3b). Conversely, the OsMED25-interacting OsARFs did not interact with mutated osmed25 protein in both yeast and plant cells (Figure 3a,b). Furthermore, no mKG fluorescence was observed in plant cells using mKGN- or mKGC-empty vectors as negative controls (Supplementary Materials Figure S1).

### 2.4. Auxin Responses in osmed25 Mutants

**Figure 4:**
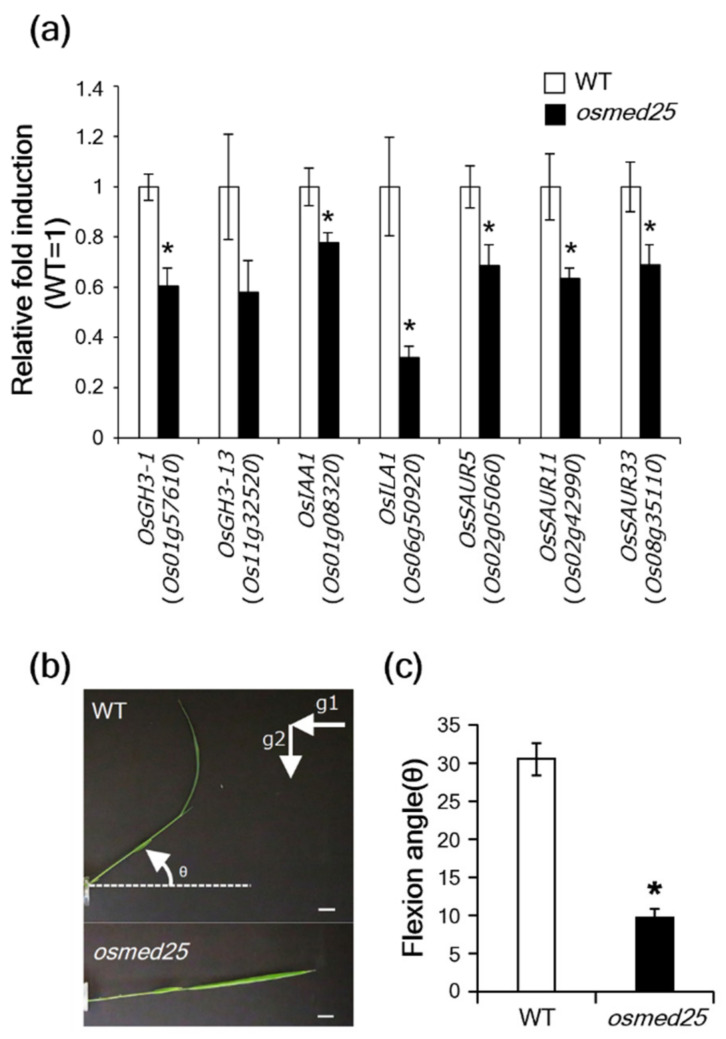
Phenotypic characterization for auxin in *osmed25* mutants. (**a**) Expression of auxin-responsive genes in *osmed25* mutants. Values are means ± SE (*n* = 6). Data were analyzed using Student’s *t*-test. Asterisks indicate significant differences from WT plants at *p* < 0.05. (**b**) Shoot gravitropism of WT and *osmed25* mutants. Rice seedlings were photographed 24 h after the start of gravity treatments (g2). Scale bars = 10 mm. (**c**) The shoot curvature angles to the horizontal direction are measured 24 h after the start of gravity treatments. Values are means ± SE (*n* = 26). Data were analyzed using Student’s *t*-test. The asterisk indicates a significant difference from WT plants at *p* < 0.05.

Some genes of the Gretchen Hagen 3 (GH3) family, which encode IAA-amino synthases, and a repressor of auxin signaling, OsIAA1, have been identified as OsARF19-responsive genes in rice [34]. Additionally, rice INCREASED LEAF ANGLE1 (OsILA1), which encodes a Raf-like mitogen-activated protein kinase kinase kinase, has been identified as an OsARF6/17-responsive gene in rice [35]. Therefore, we compared the expressions of *OsGH3-1* (*Os01g57610*), *OsGH3-13* (*Os11g32520*), *OsIAA1* (*Os01g08320*), and *OsILA1* (*Os06g50920*) in WT plants and *osmed25* mutants. In addition, we compared the expressions of the auxin-responsive small auxin-up RNA genes (*OsSAUR*s) such as *OsSAUR5* (*Os02g05060*), *OsSAUR11* (*Os02g42990*), and *OsSAUR33* (*Os08g35110*) in both plants. All of the tested genes tended to be downregulated in *osmed25* mutants in comparison to WT plants (Figure 4a).

The auxin was reported to positively regulate shoot gravitropism in rice [36]. The shoot gravitropic responses of *osmed25* mutants were significantly reduced (Figure 4b,c).

### 2.5. Auxin-Mediated Suppression of Xoo Resistance in osmed25 Mutants

**Figure 5:**
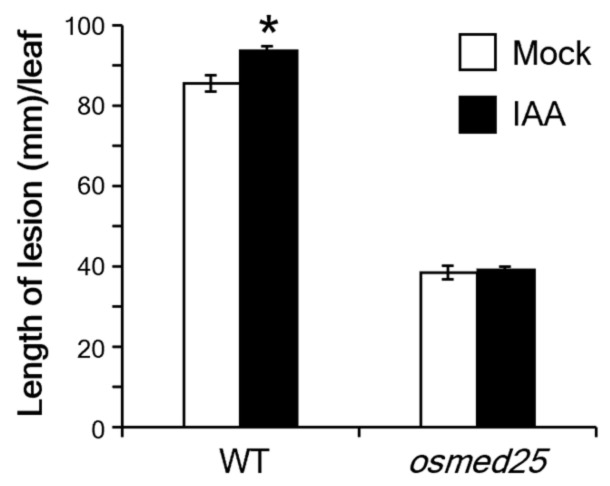
Lengths of lesions on fifth leaf blades 16 days after inoculation with *Xoo* after pretreatment with 100 nM IAA for 24 h in *osmed25* mutants. Values are means ± SE (*n* = 8). Data were analyzed using Student’s *t*-test. The asterisk indicates a significant difference from the control at *p* < 0.05.

To determine whether OsMED25 was involved in auxin-mediated suppression of *Xoo* resistance, we performed a resistance test on *osmed25* mutants after treatment with IAA. Following IAA treatment for 24 h, the plants were inoculated with virulent *Xoo*. Lengths of blight lesions were recorded at 16 days after inoculation. We found that the lengths of blight lesions in IAA-treated and -untreated *osmed25* mutants were comparable (Figure 5). However, the lengths of blight lesions among WT plants were significantly longer in IAA-treated than in IAA-untreated plants (Figure 5).

## 3. Discussion

In our previous studies, we consistently observed that JA exerts beneficial effects regarding *Xoo* resistance in rice [5,7,12,13,14,15,16,17,18,23,24,25,37]. In the present study, however, we observed that the JA-insensitive *osmed25* mutants exhibited strong resistance to *Xoo*. We revealed that the expression of OsMYC2-mediated defense signaling tended to be downregulated due to the loss-of-function of OsMED25 while the expressions of some OsMYC2-independent defense-related genes such as *Bowman–Birk proteinase inhibitors* and *OsPrx26* were constitutively upregulated in *osmed25* mutants. These results suggest that OsMED25 negatively regulates part of OsMYC2-independent defense signaling by forming complexes with uncharacterized TFs in rice. Identification of JA-mediated TFs regulated by OsMED25 is needed to comprehensively elucidate JA signaling in rice.

Transgenic rice plants overexpressing a *Bowman–Birk proteinase inhibitor* gene exhibited increased resistance to *Xoo* [38], suggesting that constitutive upregulation of some Bowman–Birk proteinase inhibitors in *osmed25* mutants may result in increased resistance to *Xoo*. Rice class III peroxidases such as OsPrx38 and OsPrx114 play an important role in *Xoo* resistance by producing lignin, which is essential for the thickening of secondary cell walls [18,20]. OsPrx114 (PO-C1) is strongly induced by inoculation with avirulent *Xoo* and is secreted to the xylem lumen and walls from the xylem parenchyma cells in rice [20]. *Xoo* contacts living cells such as xylem parenchyma cells through the pit membranes separating the xylem lumen from the associated xylem parenchyma cells. Inoculation of an avirulent *Xoo* strain triggered the thickening of xylem secondary walls and reduced the pit diameter, thus resulting in the decreased *Xoo* access to the xylem parenchyma cells [20]. Lignin is an essential component of the plant response leading to the thickening of the xylem secondary walls. Therefore, lignin production by Prxs plays an important role in the rice defense response to *Xoo* [20]. This was supported by the increased lignin content in the *osmed25* mutants, suggesting that OsPrx26 may be involved in the lignin biosynthesis of rice. Further studies such as analysis using transgenic rice plants overexpressing *Bowman–Birk proteinase inhibitors* or *OsPrx26* are needed to clarify their respective effects in relation to *Xoo* resistance in rice.

In the present study, we also revealed that OsMED25 selectively interacts with OsARFs. In particular, OsMED25 interacted with OsARF6, OsARF17, OsARF19, OsARF21, and OsARF25, and further positively regulated the expression of auxin-responsive genes, suggesting that OsMED25 is involved in the regulation of auxin signaling by forming complexes with specific OsARFs. Furthermore, we revealed that loss-of-function of OsMED25 adversely affected shoot gravitropic response as shown in *osmed25* mutants, suggesting the presence of OsMED25/OsARFs-dependent auxin responses in rice. OsMED25 contains a von Willebrand factor type A domain, a non-conserved middle domain, an activator-interacting domain (ACID), and a glutamine-rich domain [26]. The mutation site of the osmed25 protein is in the ACID, which is presumed to interact with TFs such as OsMYC2 [26,30]. In the present study, the ACID region was found to be important for the interaction between OsMED25 and OsARFs. All tested OsARFs contained a conserved N-terminal DNA-binding domain, a non-conserved middle region, and a C-terminal dimerization domain, regardless of the interaction with OsMED25, and belong to the same subgroup [33,39]. We did not find any characteristic region present in OsMED25-interacting OsARFs but did find a characteristic region present in OsMED25-noninteracting OsARFs. Three-dimensional analysis of OsARFs may provide further insight into the interactions between OsMED25 and OsARFs.

Auxins adversely affect *Xoo* resistance in rice [3]. Treatment of IAA, which is the main auxin of rice, promotes susceptibility to *Xoo* in rice [32]. In the present study, we found that the auxin-mediated suppression of *Xoo* resistance did not occur in *osmed25* mutants as shown by the downregulated expression of auxin-responsive genes, indicating that *osmed25* mutants exhibit an auxin-insensitive phenotype regarding auxin-mediated *Xoo* resistance. Auxin levels are reduced by overexpression of rice *NONEXPRESSOR OF PATHOGENESIS-RELATED GENES1*, *OsNPR1*, which is a positive regulator of *Xoo* resistance in rice [40]. Furthermore, transgenic rice plants overexpressing the cytochrome P450 gene *OsCYP71Z2* also exhibit increased resistance to *Xoo* through suppression of auxin biosynthesis [41]. *Xoo* produces and secretes IAA and induces auxin signaling in rice to facilitate the infection process [42]. Taken together, these results suggest that insensitivity to auxin derived from *Xoo* and suppression of auxin signaling regulated by OsMED25, in addition to aberrant JA signaling, resulted in strong resistance to *Xoo* in *osmed25* mutants.

OsMED25 also acts as a positive regulator of brassinosteroid signaling in rice, which plays a positive role in rice *Xoo* resistance [43,44]. In *Arabidopsis*, AtMED25 is also involved in the regulation of ethylene (ET) and abscisic acid (ABA) signaling [26]. In wheat, TaMED25 is involved in the regulation of ET signaling and adversely affects resistance to powdery mildew caused by the biotrophic fungus *Blumeria graminis* f. sp. *tritici* [45]. Both ET and ABA adversely affect *Xoo* resistance in rice [3]. Since OsMED25 is a multifunctional protein, it is involved in diverse hormone signaling pathways by forming complexes with unknown TFs and other mediator proteins in rice. Further experimental analyses are needed to clarify the regulation of hormone crosstalk through OsMED25 in rice.

## 4. Materials and Methods

### 4.1. Plant Materials, Auxin Treatment, and Bacterial Inoculation

The growth conditions of the rice plants (*Oryza sativa* L. ‘Nipponbare’) and *Xoo* (strain 7174) were the same as those previously described by Kashihara et al. [25]. The *Xoo* strain is virulent to Nipponbare. Fully opened fifth-leaf blades of the rice plants were inoculated using the clipping inoculation technique [46]. The lengths of the blight lesions were measured 14 or 16 days after inoculation. Treatment with 100 nM indole-3-acetic acid (IAA; Sigma-Aldrich, St. Louis, MO, USA) was performed following the method of JA treatment described by Yamada et al. [24]. To assess shoot gravitropic responses, 10-day-old seedlings were placed horizontally under dark conditions at 25 °C for 24 h.

### 4.2. Reverse Transcription-Quantitative PCR (RT-qPCR)

Total RNA was extracted from the fourth leaf blades using TRIzol reagent (Thermo Fisher Scientific, Witham, MA, USA). RT-qPCR was performed using TB Green Premix *Ex Taq* (Takara Bio, Kusatsu, Japan) in a Thermal Cycler Dice TP850 System (Takara Bio) according to the manufacturer’s instructions. Four leaf blades were used per replicate. Analysis of the obtained data was performed following the method of Gomi et al. [47]. Sequences of gene-specific primers used for RT-qPCR are shown in Supplementary Materials Table S1.

### 4.3. Measurement of Lignin Content

The lignin content of fully opened fourth leaf blades from three-weeks-old rice plants was determined as described by Kashihara et al. [25]. Five leaf blades were used per replicate.

### 4.4. Yeast Two-Hybrid System

The MATCHMAKER yeast two-hybrid system (Takara Bio, Kusatsu, Japan) was used, and yeast strain AH109 was used, as previously described by Suzuki et al. [30]. cDNAs of *OsMYC2* (*Os10g42430*), *OsARF6* (*Os02g06910*), *OsARF11* (*Os04g56850*), *OsARF16* (*Os06g09660*), *OsARF17* (*Os06g46410*), *OsARF19* (*Os06g48950*), *OsARF21* (*Os08g40900*), and *OsARF25* (*Os12g41950*) were ligated into the pGADT7 vector. cDNAs of *OsMED25* (*Os09g13610*) and mutated *osmed25* were ligated into the pGBKT7 vector.

### 4.5. Bimolecular Fluorescence Complementation (BiFC) Assay

The Kusabira Green (mKG) system (MBL, Nagoya, Japan) for the BiFC assay was used [18]. To visualize the nuclei, rice histone H4 (*Os10g39410*) was fused with DsRed as previously described by Onohata and Gomi [18]. The constructed vectors were expressed in onion epidermal cells using a particle bombardment system (PDS-1000/He; BioRad, Hercules, CA, USA) following the method of Kim et al. [48]. We used a KEYENCE BIOREVO BZ-9000 microscope (KEYENCE, Osaka, Japan) to observe mKG and DsRed fluorescence. The conditions were the same as those previously described by Onohata and Gomi [18].

## Data Availability

All data generated or analyzed during this study are included in this published article and its Supplementary Materials.

## Supplementary Materials

The following supporting information can be downloaded at https://www.mdpi.com/article/10.3390/plants11121601/s1; Table S1. Sequences of gene-specific primers used for RT-qPCR; Figure S1. BiFC experiments using empty vectors as negative controls in plant cells.
