# Supplementary material for: The Mutation of Rice MEDIATOR25, OsMED25, Induces Rice Bacterial Blight Resistance through Altering Jasmonate- and Auxin-Signaling
Source: Plants (Basel). 2022 Jun 17;11(12):1601. doi: 10.3390/plants11121601 (PMC9229619; doi:10.3390/plants11121601)
Supplement: Supplementary file 1 [file plants-11-01601-s001.zip › Supplementary Materials/Supplementary Materials Figure S1.pdf]

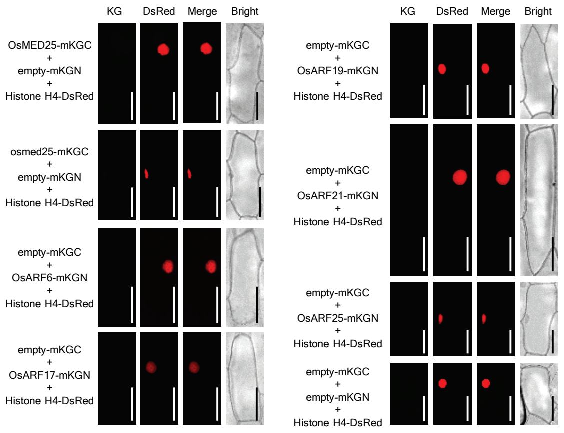

Figure S1. BiFC experiments using empty vectors as negative controls in plant cells. From left to right, the images shown are: KG, fluorescence images of KG protein; DsRed, fluorescence images of DsRed protein; Merge, overlap KG images and DsRed images; Bright, light-microscopy images. Scale bars = 100 µm.
