# Supplementary material for: The Mutation of Rice MEDIATOR25, OsMED25, Induces Rice Bacterial Blight Resistance through Altering Jasmonate- and Auxin-Signaling
Source: Plants (Basel). 2022 Jun 17;11(12):1601. doi: 10.3390/plants11121601 (PMC9229619; doi:10.3390/plants11121601)
Supplement: Supplementary file 1 [file plants-11-01601-s001.zip › Supplementary Materials/Supplementary Materials Table S1.pdf]

Table S1. Sequences of gene-specific primers used for RT-qPCR			
**Accession number**	**Gene name**	**Sense primer (5'- 3')**	**Antisense primer (5'- 3')**
Os03g03500	Resistance protein	GTAGCCCTACGTACATTTTCTCG	ATGCAGTATACACATCGGTTCTC
Os10g08780	Lipid transfer protein	GCCTACTGCCTCCAAGTTATG	GGACAATGAGCAGTATACATCAGG
Os07g48030	OsPrx112	GTAACGAGTTGCCATGCAAAG	AGCACTTCACTTGGTTAACACGAC
Os10g36100	Lipid transfer protein	TTCTGCCAGTACGCGAAAG	AGTTGGGAAAGGGGATGC
Os10g40420	Lipid transfer protein	TGTTAATTGCTTTCGGTGTCG	TGTCACTCACTCAAGCACAGG
Os02g50040	Beta-1,4-glucanase	CGAATCGGGTGATTATTGC	GCTGCAGTTAACTATACTTTGTTGG
Os10g05600	Thaumatin-like protein	ATGTCGGCGCCATTGTAG	CCCCTACAGTACAACAACAAAGC
Os01g58730	Beta-1,3-glucanase	GACGCTCCATGGATTTGG	ATCAGAAATGCAGCGGAATC
Os01g03320	Bowman–Birk proteinase inhibitor	GTGTGTTCTAGCTTGTTCGTATTCG	CACGCATACCAACATCAAACCAC
Os01g71340	Beta-1,3-glucanase	ACGAGACGGAGAGGCACTTC	TCGATCCCTTCTCAGAACAATCTTC
Os01g03360	Bowman–Birk proteinase inhibitor	CGTTCGATCATTCAGAGTTGG	CCTCATGGTCCACACAAGC
Os01g03310	Bowman–Birk proteinase inhibitor	TCTGCCGTGACTCCTTCAC	CGATGAGCCTCTCTGCTTG
Os10g02070	OsPrx126	GGCAACCAGCACAAGAGCAG	TCGTAGAAATCGTCGGATAACTG
Os12g38170	Thaumatin	AATTACTCCGGTTGGCCC	GCGCACGTACAAACATAAGG
Os02g14170	OsPrx26	ATCAGGCTCGGGAACATCG	TATGAACAATTTGCGCGCGG
Os03g31510	Proteinase inhibitor	GTCGTGTACGAGCAGTCGTG	AGGGAGGGATGGATCAGTG
Os01g57610	OsGH3-1	CGGGAACAAGCAATGGAAC	CAGATCATCACCCTCTAGCTTCAA
Os11g32520	OsGH3-13	TGTGTAATGTCAAACGTTGCTCAT	TGATTCATAAAGAACACTGCTCGTATT
Os01g08320	OsIAA1	GCGTGTCGTCAGGCTATATG	ACTCCAAAGCAAGACCATCG
Os06g50920	OsILA1	AAGAACACCAGCTGAACACC	GTTCGTCAAGCAAGCAGCAA
Os02g05060	OsSAUR5	GAAGGCAGAGGAGGAGTTTGG	CAAACACCTCAACATCACATGGA
Os02g42990	OsSAUR11	ACGTGTCATGTGTGCTCGAT	GCAAAGATGCGAACACGTTG
Os08g35110	OsSAUR33	AGTGAAGAGTACGAAGCGGC	ATTGGACCGATGGGCATGTT
Os11g06390	Actin	GAGTATGATGAGTCGGGTCCAG	ACACCAACAATCCCAAACAGAG
